# Selected Properties of Two Alternative Plant Fibers: Canola and Sweet Clover Fibers

**DOI:** 10.3390/ma15227877

**Published:** 2022-11-08

**Authors:** Vahid Sadrmanesh, Ying Chen

**Affiliations:** Department of Biosystems Engineering, University of Manitoba, Winnipeg, MB R3T 5V6, Canada

**Keywords:** canola, sweet clover, fiber, property

## Abstract

Identifying sustainable resources of natural fibers is essential due to their high demand in industrial applications such as automotive and biomedical materials. Two alternative fibers obtained from canola and sweet clover stalks were characterized for their properties using energy dispersive X-ray spectroscopy (EDS), Fourier transform infrared spectroscopy (FTIR), X-ray diffraction (XRD), thermogravimetric analysis (TGA), contact angle, and tensile test. Hemp and flax fibers, both in use as industrial fibers, were also characterized as conventional fibers. Results showed that all the fibers had the same chemical elements (carbon, oxygen, magnesium, and potassium) and chemical bonds. The crystallinity index for the alternative fibers ranged from 62 to 71%, which was close but lower than the conventional fibers (82% for hemp and 80% for flax). The thermal stability of the alternative fibers was around 220 °C, close to the conventional fibers (230 °C). The alternative fibers had contact angles of less than 90°, showing high surface energy. Since the alternative fibers had a low Young’s modulus and tensile strength (5.57–8.52 GPa and 57.45–71.26 MPa, respectively), they are suitable for some specific applications in the biomedical industry. In contrast, conventional fibers are suitable where a higher stiffness and strength is required.

## 1. Introduction

The replacement of synthetic-based fibers with natural fiber products has considerably increased due to growing ecological, economic, and social awareness, as well as a governmental emphasis on environmental effects and sustainability. Furthermore, the recycling process of natural fibers generates low-toxicity fume emissions compared to that of synthetic fibers [1]. Natural fibers are obtained from three major sources: plants, minerals, and animals [2]. Plant natural fibers are easier to recycle when compared to mineral and animal fibers. Other attractive properties of plant-based natural fibers are a low density with an acceptable tensile strength (300–1200 MPa) and stiffness (30–70 GPa) [3].

Bast fibers are one of the most popular plant fibers. They are extracted from the stem of fibrous plants. Their main chemical compositions are cellulose, hemicellulose, lignin, and pectin [4]. The percentage of these compositions, which significantly affect the technical properties of bast fibers, are different from fiber to fiber depending on the age, fiber source, climate, harvesting time, and extraction process conditions [5]. For example, fibers with a high cellulose percentage have a high tensile strength and Young’s modulus but a low strain. As another example, fibers with a high percentage of cellulose and lignin are more resistant to degradation and are therefore associated with high thermal stability.

Combining bast fibers with polymers generates biocomposite materials. The characterization of biocomposites specifies their application [6]. For instance, biocomposites with high-impact properties and favorable tensile strengths are appropriate where there is more impact stress. In contrast, biocomposites with high tensile strengths and low-impact properties are appropriate for the furniture industry. The characteristics of a biocomposite depend on the properties of the plant fiber. Therefore, understanding the properties of alternative plant fibers associated with acceptable characteristics is essential in developing high-performance biocomposites. Some of the alternative fibers already introduced are buriti fiber [7], okra fiber [8], artichoke fiber [9], ferula fiber [10], Borassus fiber [11], Conium maculatum fiber [12], *Coccinia grandis*. L fiber [13], and manau rattan fiber [14]. However, there are still many other alternative natural fibers to explore.

Two alternative fibrous plants in Canada are canola (Brassica Napus) and sweet clover (Melilotus). Canada is one of the leading countries in the world in producing canola seeds, with a total canola harvested area of 9.27 Mega hectares in 2017 [15]. After the seeds are harvested, a large amount of canola residues remains in the field. The residues would contribute to the soil’s organic carbon, but sometimes they are considered waste when there is excessive canola stalk in the field. Extracting fiber from these residues provides an alternative residue management method for farmers. The wild sweet clover plant can also be found Canada-wide. This plant is drought-resistant, can be grown in northern latitudes, and is compatible with saline land [16]. 

This study aimed to investigate the possibility of using canola and sweet clover fibers for industrial purposes. Most used techniques to characterize fibers are microstructural, thermal, surface, and mechanical analyses. The microstructure of the fibers affects their physical and mechanical properties which are used to predict the performance of the developed bioproducts [17]. The thermal behavior of natural fibers is of importance since they undergo several high-temperature processes during the bioproducts manufacturing process. Besides, applying a high temperature to the lignocellulosic fibers results in undesirable properties of the final products. Contact angle, which is a good indicator of the surface energy of the fibers, is also a good scale of interfacial shear strength (IFSS) controlling the performance of the developed bioproducts. The mechanical properties of natural fibers strongly control the mechanical properties of the bioproducts [5]. Therefore, the objective of this study was to investigate the microstructural, thermal, surface, and mechanical properties of canola and sweet clover fiber in comparison to the traditional fibers of hemp and flax. The characteristic investigations included energy dispersive X-ray spectroscopy (EDS), Fourier transform infrared spectroscopy (FTIR), X-ray diffraction (XRD), thermogravimetric analysis (TGA), contact angle, and tensile properties analyses. 

## 2. Materials and Methods

### 2.1. Types of Plant Fibers

**Figure 1:**
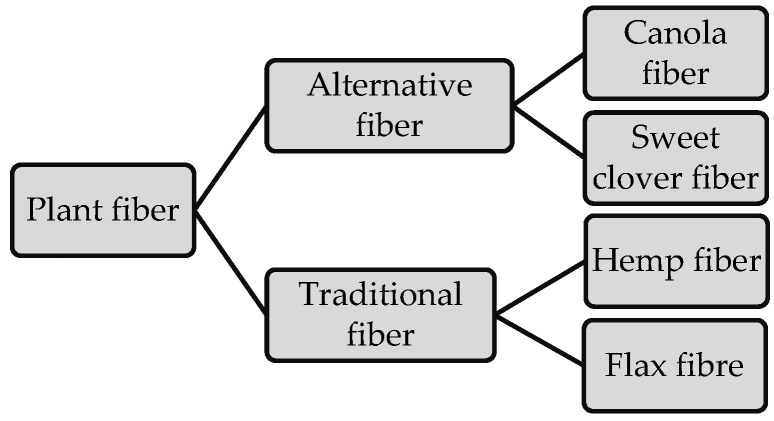
Types of fibers characterized.

Four plant fibers were selected for this study (Figure 1). Two of them were alternative fibers from canola and sweet clover. They were compared with the other two traditional fibers from hemp and flax. Canola plants (Red River 1861 type) were obtained from Glenlea Research Center, University of Manitoba; sweet clover plants (wild-type) were obtained from a farm in the south of Winnipeg, MB, Canada; hemp plants (68 Alyssa-type) were provided by Baker Farms, Dauphin, MB, Canada; and Linseed flax plants (sorrel variety) stalks were provided by the Composite Innovation Centre, MB, Canada, hand-harvested from various locations in Manitoba, Canada. All plants were hand-harvested in August 2014.

**Figure 2:**
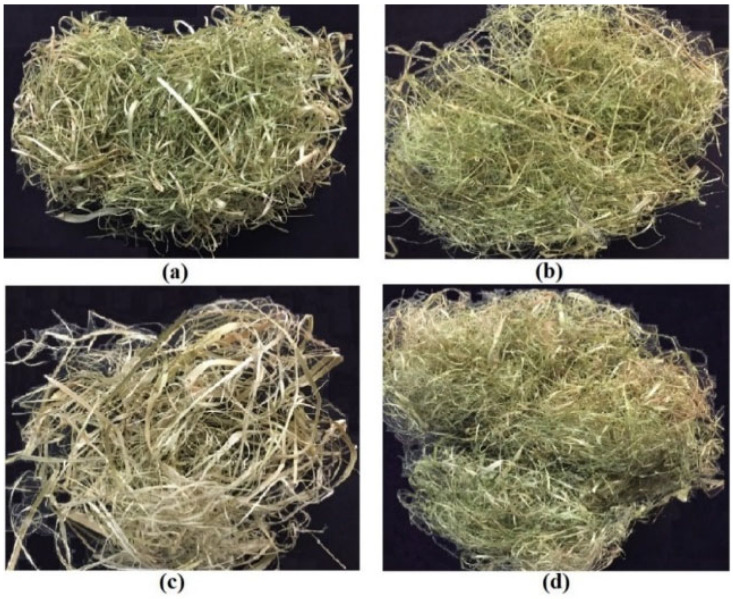
(**a**) Canola fiber; (**b**) Sweet clover fiber; (**c**) Hemp fiber; and (**d**) Flax fiber.

The plant stems were cleaned from the leaves, sub-branches, seeds, etc. The main branch of the stalks was cut into small segments. Then, in order to remove contaminations from the segments, they were washed with tap water twice and reverse osmosis water once and air-dried for 15 days. The air-dried samples were then placed in an oven for two hours at 135 °C to determine the moisture content of the segments. The moisture content of the canola, sweet clover, hemp, and flax stalks at the time of fiber separation was 4.9%, 4.1%, 3.3%, and 3.7% (dry based), respectively. Finally, the fibers were hand peeled, air-dried, and stored in paper bags for further analysis [18]. Figure 2 shows all of the extracted fibers.

### 2.2. Fiber Characterization 

#### 2.2.1. Chemical Compositions 

The main chemical compositions, including cellulose, hemicelluloses, and lignin were measured for each of the four fibers. The measurements were performed using neutral detergent fiber (NDF) and acid detergent fiber (ADF) [16].

#### 2.2.2. Microstructural Characterization 

The microstructural properties of the obtained fibers were studied using energy dispersive X-ray spectroscopy (EDS), Fourier transform infrared spectroscopy (FTIR), and X-ray diffraction (XRD) tests illustrated as follows. 

The EDS is an analytical approach to measure the percentage of different elements such as C and O along with Na, Al, Si, and Mg on the fiber surface. However, EDS is unable to identify H, which shows the main constituents of natural fibers. An FEI Nova NanoSEM 450 was used to take scanning electron microscopy images for the EDS analysis. The accelerating voltage of the instrument was set between 10 and 15 kV, the spot size was adjusted to 30 µm, and the working distance was kept to 10 mm. The pictures were taken at a scanning speed of four to obtain a higher image quality. From each type of fiber, three samples were selected, the distribution of the elements was measured four times, and the average values were reported. 

The FTIR tests in attenuated total reflection mode were performed to identify the chemical functional groups and the types of bonds in the fibers. A fiber sample was placed against a diamond, and then the infrared beam interacted with the sample at the interface. An evanescent wave penetrated the sample to a shallow depth, while the radiation underwent total internal reflection at the crystal surface, and the absorption of this component produced the infrared spectrum [19]. The IR spectrum of the samples was recorded in the 4000–650 cm^−1^ region with 32 scans and a resolution of 4 cm^−1^. The number of replications for each type of fiber was four.

The crystallinity index of the studied fibers was determined using XRD analysis. A Bruker D8 DaVinci diffractometer with CuKα radiation was used to measure the crystallinity index of the samples. After the sample was prepared and placed into the machine, the X-ray detector rotated over a range of 2ϴ values from 10° to 50° at a scanning speed of 0.03 mm s^−1^, a current of 40 mA, and a voltage of 40 mV.

#### 2.2.3. Thermal Characterization 

The thermal behavior of the samples was analyzed using a Perkin Elmer TGA 7 thermogravimetric analyzer. The samples were placed in an alumina pan and heated from room temperature to 600 °C at a rate of 2 °C min^−1^ while maintaining a static argon flow of 150 mL min^−1^. The TG graph was plotted as the percentage of mass loss of the fiber at various temperatures. A DTG graph was also generated for the rate of change in weight at various temperatures by using the first derivative values of the mass losses.

#### 2.2.4. Contact Angle Measurement

To measure the contact angle of the fibers, a Sigma 700 Attension force tensiometer (Biolin Scientific) was employed. Prior to performing the tests, all of the samples were dried for 3 h at 80 °C. The Washburn capillary rise method was selected to estimate the contact angle. Since the edge of the fibers were not spherical, the first 0.1 mm of each sample was ignored to decrease errors. The immersion speed for both advancing (penetration of the fiber into water) and receding (recession of the fiber from water) was set to 5 mm min^−1^, and the contact angle was estimated.

#### 2.2.5. Mechanical Characterization

The tensile properties of the samples were measured according to ASTM Standard D3822 [20]. A Lloyd LS5 electromechanical testing system (Ametek Inc., Largo, FL, USA) was used. The Lloyd system consisted of a frame with a movable crosshead, a load cell attached to the crosshead, a pair of clamps, a drive system for the crosshead, and controller software (Nexygen, version 4.0). The bottom clamp was a stationary grip attached directly to the base of the frame, while the top clamp was connected to the load cell (5 kN capacity) which itself was connected to the crosshead. 

To measure the tensile properties of the fibers, their cross-sectional area was needed. The cross-sectional areas of plant fibers are closer to rectangular than circular [21]. From each fiber type, 20 fiber samples were randomly chosen. They were cut into 25 mm long specimens. Prior to the tensile tests, the width and thickness of the specimens were measured. In the measurement, a fiber was attached to two aluminum cube blocks covered by a double-coated carbon conductive tape (Ted Pella, Inc. USA). Nine measurements along approximately 10 mm of the middle section of the fiber were performed. For the tensile test, a fiber specimen was attached to a thin cardboard frame using a fabric adhesive which was secured using the clamps [22]. After cutting the sides of the cardboard frame and setting a 2 N preload to remove any slack, the drive system moved the crosshead up at a loading rate of 25 mm min^−1^ until the fiber specimen failed. The Lloyd machine’s Nexygen software (version 4.0) recorded the tensile load and machine extension for each test and calculated the tensile properties.

## 3. Results and Discussions

### 3.1. Chemical Compositions

Table 1 shows the main chemical compositions of the studied fibers. The percentage of cellulose content in the alternative fibers (canola and sweet clover) was lower than in the traditional fibers (hemp and flax). However, the percentage of lignin in the alternative fibers was higher than that in flax fibers. Sweet clover fibers had the highest percentage of hemicellulose while canola fibers had the lowest percentage [16,23].

### 3.2. Microstructure Analysis

**Figure 3:**
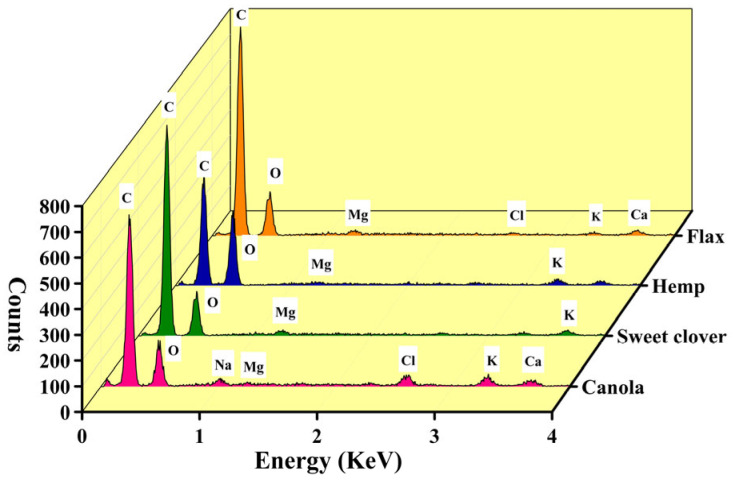
The chemical elements of the studied fibers.

The EDS analysis of the studied fibers demonstrated that, regardless of hydrogen, the main compositional elements of the canola and sweet clover fibers, like flax and hemp fiber, were carbon and oxygen (Figure 3), giving the expected results of lignocellulosic fibers [13,24]. A low percentage of elemental carbon is desirable since it gives a low non-cellulosic material. The lowest percentage of carbon, regarding both weight and atomic basis, was related to hemp, followed by sweet clover, and canola and the highest was in flax fiber. Magnesium and potassium were the other elements found in all the fibers, but canola fiber was the only fiber containing sodium.

**Figure 4:**
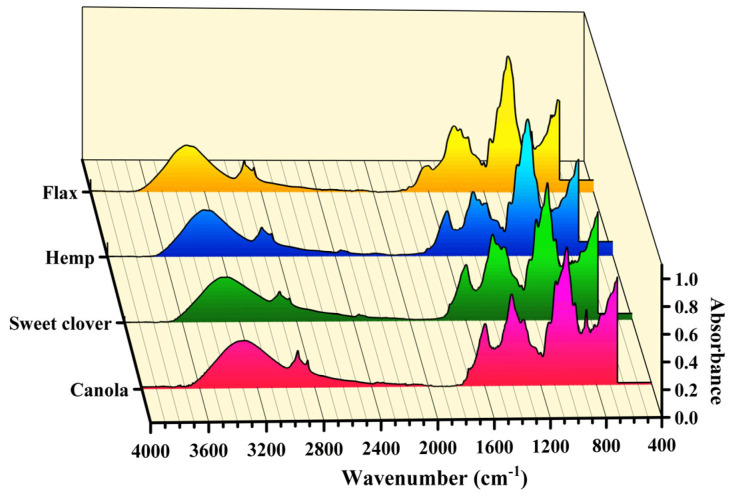
The average FTIR spectrum of the studied fibers.

Figure 4 demonstrates that the trend of the ATR–FTIR spectrum of the new fibers (e.g., canola and sweet clover) were almost similar to the traditional plant fibers (e.g., hemp and flax), proving that all of them have the same chemical bonds. The absorbance bonds at around 3400 to 3200 cm^−1^ were due to the O-H stretching via vibration, as well as the hydrogen bond of the O-H stretching vibration in cellulose, hemicellulose, lignin, and pectin [10,25]. The C-H stretching vibration in the general organic material, such as wax, produced a peak at around 2900 cm^−1^ [19]. The absorbance peaks at around 2840 cm^−1^ could be explained by the C-H stretching vibration from CH and CH2 in all chemical components of the fibers [25]. The peaks at around 1735 cm^−1^ were associated with the C=O ester bond from pectin [19] and the peak centered around 1600 cm^−1^ represents the O-H stretching group in the absorbed water [26]. The absorbance at around 1595 and 1505 cm^−1^ arose from the C=C stretching of the aromatic ring of the lignin [8,27]. The absorbance peaks at around 1475 cm^−1^ were attributed to the CH2 symmetric bending in cellulose, lignin, hemicellulose, and pectin [27,28]. The vibration of the C-H and C-O groups of the aromatic ring at an absorbance of around 1365 and 1315 cm^−1^ were because of polysaccharides [29,30]. The C-O stretching vibration of the acetyl group in lignin produced a peak at around 1235 cm^−1^ [30,31]. The absorbances at around 1155 cm^−1^ and 1105 cm^−1^ were the characteristic bonds for the C-C ring and C-O-C glycosidic ether of the polysaccharide components, respectively, which were largely cellulosic [19]. The b-glycosidic linkages between the monosaccharides of cellulose and hemicellulose generated intense peaks at around 900 cm^−1^ [8,27].

**Figure 5:**
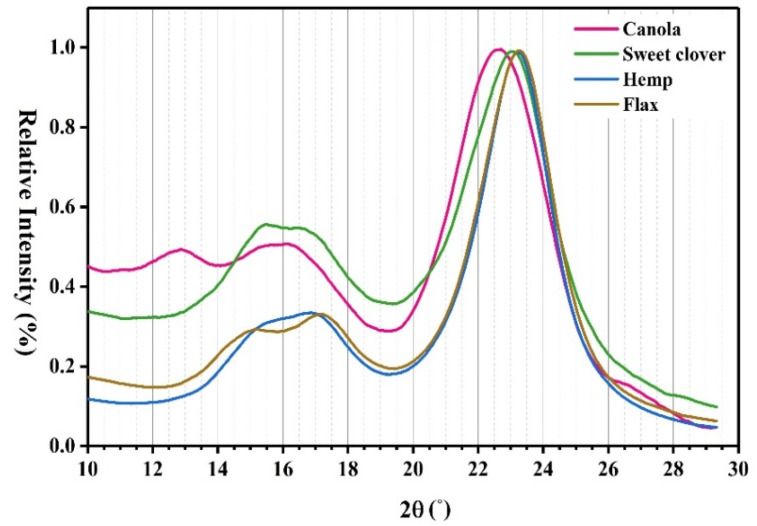
X-ray diffraction curves for the studied fibers.

The crystallinity index, which is one of the most important structural parameters, strongly influences the mechanical properties of the natural fibers such that natural fibers with a high crystallinity index have greater stiffness. Figure 5 shows the X-ray diffraction graph of the investigated fibers. The two main reflections at 18° < 2ϴ < 20° and 22° < 2ϴ < 24° correspond to the amorphous and crystalline part in the fiber, respectively. 

From a qualitative standpoint, the intensity of the crystalline parts of the fibers was almost similar but the amorphous parts of the new fibers were higher than the common fibers. It could be concluded that the structure of hemp and flax fibers was more crystalline than the canola and sweet clover fibers. To compare the fibers from a quantitative standpoint, the crystallinity index was calculated using Equation (1) [32].

(1) $$\mathrm{CI}=\frac{I_{002}-I_{\mathrm{amp}}}{I_{002}}\times 100$$

where I_002_ is the intensity of the peak around the crystalline phase, and I_amp_ is the intensity of the peak around the amorphous phase. The results showed that the crystallinity index of the canola and sweet clover fibers were 71% and 62%, respectively, which were higher than the crystallinity index of alternative plant fibers already introduced, including 52.27% for Sansevieria ehrenbergii fiber [33], 60% for Sansevieria cylindrica fiber [34], and 68% for A. officinalis L. fiber [35]. The crystallinity index of the hemp (82%) and flax (80%) fibers were the highest but in the same range as that found in the studies [12,36,37].

### 3.3. Thermal Analysis

**Figure 6:**
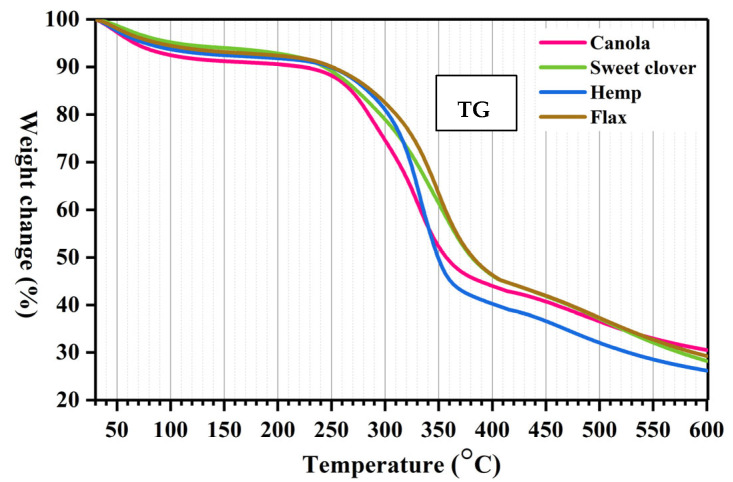

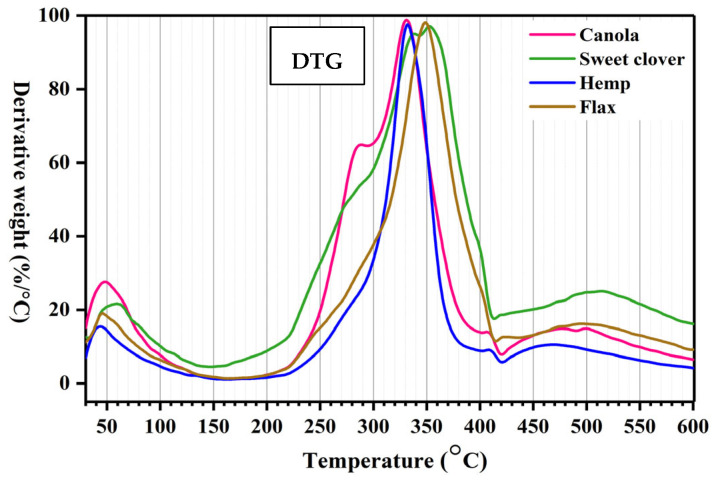
The weight loss curve (TG) and derivative curve (DTG) obtained from the TGA analysis for the studied fibers.

The TG and DTG curves of the investigated fibers showed that the thermal behavior of the new lignocellulosic fibers was similar to the hemp and flax fibers (Figure 6). The initial peaks in the DTG graph located between 30 to 100 °C were due to the vaporization of the moisture in the fiber. Hemicellulose was the first chemical composition to decompose because of its amorphous structure and position within the fibers. For the studied fibers, the range for hemicellulose decomposition was from 219 °C to 305 °C. The initial degradation temperature for the canola and sweet clover fibers, where hemicelluloses started decomposing, referring to the thermal stability of the fibers, was 220 °C and 219 °C, respectively. These values were close to the initial degradation temperature of flax (219 °C) and hemp fiber (236 °C). It is clear that the canola and sweet clover fibers had similar thermal stabilities to hemp, flax, and of other natural fibers, such as buriti fiber (150 °C) [7], okra fiber (220 °C) [8], and artichoke fiber (230 °C) [9]. Cellulose was the second chemical composition starting to decompose. The reason for the higher thermal stability of cellulose compared to hemicellulose was due to several microfibrils, which are responsible for fiber reinforcement, located in the structure of cellulose. The range where the cellulose of the studied fibers decomposed varied from 292 °C to 413 °C. The maximum degradation temperature showing the cellulose decomposition for canola, sweet clover, hemp, and flax were 331 °C, 355 °C, 334 °C, and 349 °C, respectively. Lignin provides rigid support to the fibers and supplies higher thermal stability to the fibers than hemicellulose and cellulose. Lignin decomposed from at a very low temperature to 600 °C due to its complex structural composition of aromatic rings with various branches [5]. The small peaks at temperatures ranging from 460 °C to 520 °C were due to the oxidative degradation of the charred residue. Residues of 31%, 28%, 26%, and 29% at 600 °C for canola, sweet clover, hemp, and flax fibers, respectively, were probably owing to the non-oxidizing atmosphere used in the experiment [7].

### 3.4. Contact Angle Analysis

The advancing and receding angles of the examined fibers were measured. There was a difference between the advancing and receding angles in which advancing angles for all fibers were greater than the receding angles. The reasons for this hysteresis (e.g., differences between advancing and receding angles) were the geometric and chemical heterogeneity or small-scale roughness of the samples’ surface. Table 2 shows the advancing and receding angles of the studied fibers. The contact angles for canola and sweet clover fibers, regardless of advancing or receding, were less than 90°, similar to hemp and flax fibers. A contact angle less than 90° is desirable as it shows a high surface energy and high wettability meaning that the matrix will spread over a large area of fibers, and a high IFSS is achievable. However, contact angles greater than 90° show a minimum tendency of the matrix to contact with fibers which is undesirable. The minimum advancing and receding angles (e.g., the most favorable) were for sweet clover and canola fiber at 71.63° and 29.36°, respectively, while the flax fiber had the maximum angle for both advancing (81.42°) and receding (64.96°). The reason for the different values of contact angle was due to the different structural arrangements of the chemical components of a single fiber of the studied fibers. A single fiber is constituted from primary and secondary walls. The primary wall is made of hemicellulose, pectin compounds, and glycoproteins. The secondary wall is a three-layer structure (S1, S2, and S3) which is made from cellulose, hemicellulose, and lignin. Therefore, the polarity of different fibers may vary due to varying chemical components in each section of the fiber network [5].

### 3.5. Mechanical Analysis

**Figure 7:**
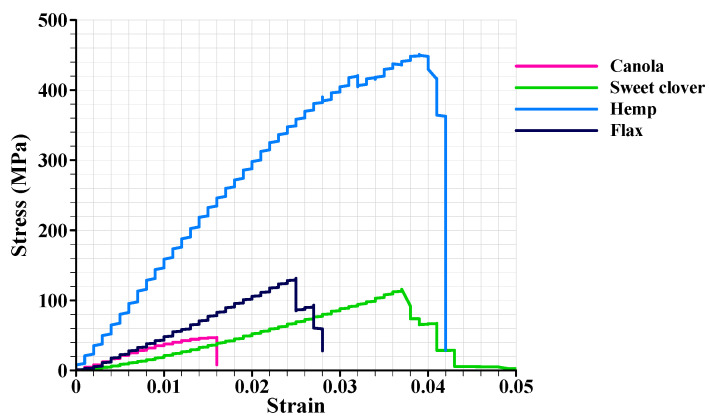
The stress–strain curves for the investigated plant fibers.

The typical stress–strain curves for all of the plant fibers are shown in Figure 7. For all the fibers, stress increased to a peak value and then suddenly dropped to zero. Most samples failed at maximum strength and behaved like brittle materials. Other studies also proved that natural fibers behave like a brittle material [9,38,39]. 

**Figure 8:**
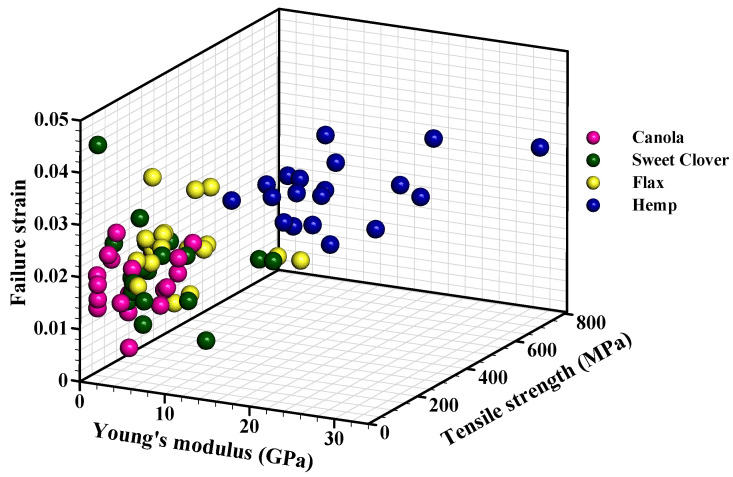
Failure strain, tensile strength, and Young’s modulus of the studied fiber bundles.

There was a high scatter in the tensile properties of the fibers, even within one specific fiber (Figure 8). This variation was due to three main factors including plant characteristics (i.e., the growing conditions, agronomic practices, harvesting time, fiber extraction method, and the presence of defects), cross-sectional area measurements, and test parameters/conditions [40]. 

**Figure 9:**
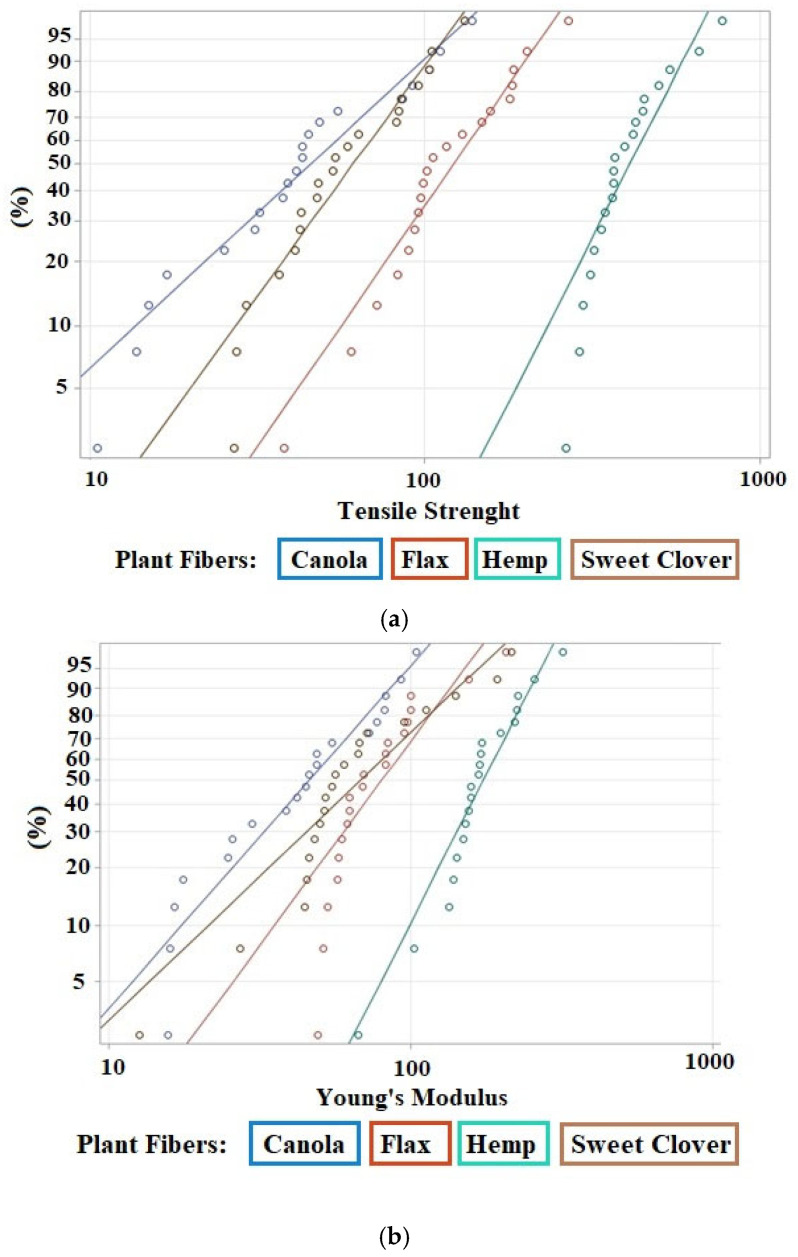
Weibull probability for mechanical properties of the studied plant fibers (*X*-axis is a log scale): (**a**) Weibull probability for tensile strength; (**b**) Weibull probability for Young’s modulus.

Where there is a large variation among data, the two-parameter Weibull distribution is used to statistically analyze the reliability of the data. Several researchers used Weibull cumulative distribution to determine the reliability of the mechanical performance of natural fibers [8,9,41,42,43]. Figure 9 presents the Weibull distribution plots for tensile strength and Young’s modulus of the investigated fibers. The results of the Weibull distribution analysis showed a reasonable approximation of the experimental data for both tensile strength and Young’s modulus.

Table 3 shows statistical parameters obtained after analyzing the data. The shape parameter which is also known as the Weibull modulus shows the variability of the data, and a higher value is more desirable. For both tensile strength and Young’s modulus, the hemp fibers had the lowest variability in data (e.g., highest shape parameter), and the highest variability (e.g., lowest shape parameter) for tensile strength was related to the canola fibers, while the highest variability for Young’s modulus was for the sweet clover fibers.

The scale parameter, which is also known as the Weibull characteristic, is the 63.2 percentile of the data. It has the same trend as the average tensile strength and Young’s modulus. The hemp fiber was the strongest and stiffest. The scale parameter for the tensile strength and Young’s modulus of the hemp fiber bundle were 456.26 MPa and 19.36 GPa, respectively. In contrast, the canola fiber bundle was the weakest in which the scale parameter for tensile strength was 57.45 MPa. The flax fiber bundle was the most flexible with a scale parameter of 9.39 GPa for Young’s modulus (Table 3). The scale parameter for the tensile strength of the hemp and flax fibers were significantly higher than for canola and sweet clover, demonstrating that hemp and flax fibers are suitable for use as reinforcement in structural materials, but canola and sweet clovers are appropriate for other applications where a low elastic modulus and normal tensile strength are required. The reason for the higher mechanical properties of the conventional fibers (hemp and flax fibers) compared to the alternative fibers (canola and sweet clover fibers) was due to the high crystalline structure of the conventional fibers. The microstructural results presented earlier in this study showed that the crystallinity index of the hemp and flax fibers was 82% and 80%, respectively, which was higher than that for canola (71%) and sweet clover fibers (62%).

The mechanical properties of the canola and sweet clover fibers are comparable to those of other natural fibers recently introduced as potential reinforcement in composites. For example, the tensile strength and Young’s modulus of abaca, alfa, coir, cotton, jute, and sisal fibers were 12, 350, 140.5, 500, 325, 460 MPa, and 41, 22, 6, 8, 37.5, 15.5 GPa, respectively [44].

## 4. Conclusions

The properties of the two alternative lignocellulosic fibers (canola and sweet clover) were compared with two traditional lignocellulosic fibers (hemp and flax). The following conclusions were drawn:

- The alternative fibers had the same microstructure as the conventional fibers. The main chemical elements, chemical bonds, and crystallinity index of the alternative fibers were highly similar among these fibers. For example, the percentage of cellulose as the main chemical component for all the fibers was in the range of 44 to 60. Besides, the main compositional elements of all the fibers were carbon, oxygen, magnesium, and potassium. The CI index of the alternative fibers was around 62–71% which was higher than for other new fibers introduced by other researchers.
- When heated, the behaviors of all the fibers were similar. The thermal stability of the alternative fibers (220 °C) was close to the conventional fibers (230 °C). This thermal stability is high enough for fibers to be used in the development of commercial bioproducts.
- The contact angles of the alternative fibers were less than 90° showing high surface energy and high wettability which leads to a high IFSS.
- Canola and sweet clover fibers were associated with a low Young’s modulus (5.57 and 8.52 GPa, respectively), and acceptable tensile strength (57.45 and 71.26 MPa, respectively), while hemp and flax fibers were associated with high stiffness (19.36 and 9.39 GPa, respectively), and high strength (456.26 MPa and 141.23 MPa, respectively).

The aforementioned characteristics of the fibers were found to be highly variable. The fibers used were obtained from certain plants grown in certain climates and soil conditions. It is expected that different growth conditions would affect the characteristics of the fiber. Caution should be taken when extending these results. One of the potential applications of canola and sweet clover fibers is in the biomedical industry, where low stiffness and acceptable tensile strength are desirable. Further research is required in this regard.

## Figures and Tables

**Table 1:** Main chemical compositions of the studied fibers.

Fiber	Cellulose (%)	Hemicellulose (%)	Lignin (%)
Canola	44	6	19.21
Sweet clover	53	16.77	13.6
Flax	60	7	27
Hemp	60.2	14.8	11.2

**Table 2:** Contact angles of the studied fibers.

Fiber	Advancing Angle (°)	Receding Angle (°)
Canola	79.17	29.36
Sweet clover	71.63	52.13
Flax	81.42	64.96
Hemp	77.36	59.89

**Table 3:** Weibull distribution parameters for mechanical properties of the studied plant fibers.

Property	Parameter	Canola	Sweet Clover	Flax	Hemp
Tensile strength, MPa	Shape parameter	1.57	2.34	2.43	3.32
	Scale parameter	57.45	71.26	141.23	456.26
	Mean	51.61	63.14	125.24	409.40
	Standard deviation	33.64	28.71	54.79	135.85
Young’s modulus, GPa	Shape parameter	1.93	1.62	2.30	3.34
	Scale parameter	5.57	8.52	9.39	19.36
	Mean	4.94	7.63	8.32	17.38
	Standard deviation	2.66	4.82	3.83	5.73

## Data Availability

Not applicable.
